# Roles of disease severity and post-discharge outpatient visits as predictors of hospital readmissions

**DOI:** 10.1186/s12913-016-1814-7

**Published:** 2016-10-10

**Authors:** Hao Wang, Carol Johnson, Richard D. Robinson, Vicki A. Nejtek, Chet D. Schrader, JoAnna Leuck, Johnbosco Umejiego, Allison Trop, Kathleen A. Delaney, Nestor R. Zenarosa

**Affiliations:** 1 Department of Emergency Medicine, Integrative Emergency Services, John Peter Smith Health Network, 1500 S. Main St., Fort Worth, TX 76104 USA; 2 Institute for Health Aging, Center for Alzheimer’s and Neurodegenerative Disease Research, University of North Texas Health Science Center, 3500 Camp Bowie Blvd., Fort Worth, TX 76107 USA

**Keywords:** Hospital readmission, APR-DRG, Post-discharge visit, Prediction

## Abstract

**Background:**

Risks prediction models of 30-day all-cause hospital readmissions are multi-factorial. Severity of illness (SOI) and risk of mortality (ROM) categorized by All Patient Refined Diagnosis Related Groups (APR-DRG) seem to predict hospital readmission but lack large sample validation. Effects of risk reduction interventions including providing post-discharge outpatient visits remain uncertain. We aim to determine the accuracy of using SOI and ROM to predict readmission and further investigate the role of outpatient visits in association with hospital readmission.

**Methods:**

Hospital readmission data were reviewed retrospectively from September 2012 through June 2015. Patient demographics and clinical variables including insurance type, homeless status, substance abuse, psychiatric problems, length of stay, SOI, ROM, ICD-10 diagnoses and medications prescribed at discharge, and prescription ratio at discharge (number of medications prescribed divided by number of ICD-10 diagnoses) were analyzed using logistic regression. Relationships among SOI, type of hospital visits, time between hospital visits, and readmissions were also investigated.

**Results:**

A total of 6011 readmissions occurred from 55,532 index admissions. The adjusted odds ratios of SOI and ROM predicting readmissions were 1.31 (SOI: 95 % CI 1.25–1.38) and 1.09 (ROM: 95 % CI 1.05–1.14) separately. Ninety percent (5381/6011) of patients were readmitted from the Emergency Department (ED) or Urgent Care Center (UCC). Average time interval from index discharge date to ED/UCC visit was 9 days in both the no readmission and readmission groups (*p* > 0.05). Similar hospital readmission rates were noted during the first 10 days from index discharge regardless of whether post-index discharge patient clinic visits occurred when time-to-event analysis was performed.

**Conclusions:**

SOI and ROM significantly predict hospital readmission risk in general. Most readmissions occurred among patients presenting for ED/UCC visits after index discharge. Simply providing early post-discharge follow-up clinic visits does not seem to prevent hospital readmissions.

## Background

Thirty-day all-cause hospital readmission is a fundamental patient outcome measurement reported publicly in the US. It is also a metric used by the Centers for Medicare and Medicaid Services (CMS) as a financial incentive to drive patient care quality improvement initiatives among participating institutions, medical groups, and individual providers [1–3]. Many studies have focused on identification of patients at high risk for readmission, however the strength of predictors varies in the literature according to the different diseases and selected populations studied [4, 5]. Available data suggest in-patient length of stay, age, and lack of post-hospital follow-up visits are independent risk factors predicting hospital readmissions [6–8]. Other risks such as gender, patient psychosocial status, and history of substance abuse have also been reported albeit these indices have provided inconsistent results [9–12]. These risk factors (age, in-patient length of stay, comorbidities, and/or psychosocial status) were reported not only in adult patient populations but also in pediatric patients. Two recent large pediatric studies on hospital readmissions yielded similar results [13, 14]. Additionally, multifactorial risks are incorporated into different scoring systems previously reported in the literature. Pooling a variety of risk factors and adjusting their weights to predict readmission of specific diseases may contribute to data discrepancies. For example, the LACE index has been used to predict the risk of readmission in both medical and surgical patients by calculating a composite score that includes length of stay (“L”), acuity of admission (“A”), comorbidities (“C”), and the number of ED visits over the past 6 months (“E”) [15]. Higher LACE scores predict a higher likelihood of patient readmissions [15]. Another method using the HOSPITAL score was derived to identify high-risk readmission patients [16]. While this method had fair discriminatory power [16], equipoise exists regarding the accuracy of using these scoring systems to predict unplanned readmissions in other studies [17, 18].

Recently, the All Patient Refined Diagnosis Related Group (APR-DRG) was developed by 3 M Health Information Systems in a joint effort with National Association of Children’s Hospitals and Related Institutions [19]. Its initial purpose was to properly determine the appropriate value of care for higher acuity patients thereby providing a better model for predicting resource needs [20]. APR-DRG is a clinical model and is disease specific. Each APR-DRG is subdivided into four severity of illness (SOI) and risk of mortality (ROM) subclasses (e.g. minor, moderate, major, and extreme). A higher level of SOI/ROM indicates more severe illness and higher mortality resulting in more medical care and hospital resource needs. Each patient is initially assigned an APR-DRG score based on their primary disease and then subsequently their appropriate level of SOI and ROM are calculated. SOI is calculated based on patient age, primary diagnosis along with severity of secondary diagnoses. It takes patient comorbidities into consideration with respect to disease severity and could place an initial non-severe primary disease into an extremely severe illness category if the patient is an elderly person with multiple severe comorbidities. Therefore, SOI determines overall patient illness severity according to the extent of physiological decomposition or organ system loss of function. ROM estimates the likelihood of dying. Several recent studies found ROM and SOI are useful in predicting hospital readmissions [10, 21], whereas others still question the validity of these tools [22]. Given the challenge that greater variety and complexity impart to predicting hospital readmissions, it seems critically important to identify multiple risks for readmission along with ROM and SOI to better determine the accuracy of hospital readmission predictions.

Post-discharge hospital follow up visits are also considered another important factor in readmission prevention [23]. Park et al. [24] examined the Veterans Affairs Hospital System and reported that providing only one post-discharge clinic visit significantly reduces readmission rates by 9 %. Tuso et al. [25] also found that providing a transitional care bundle including a timely primary care physician follow-up outpatient visit significantly reduced hospital readmissions in the state of California. Some studies show similar results among patients with different diseases [26, 27]. In contrast, other studies show that simply scheduling post-discharge clinic visits does not prevent hospital readmissions in different patient populations [28, 29]. Although complicated discharge bundle programs that include post-discharge follow-up clinic visits, phone interventions, and/or assistance with medication adjustments may increase patient satisfaction scores, these augmented services are not robust enough to prevent hospital readmissions [30, 31]. Considering the important role of post-discharge follow-up visits in the continuity of care model, we sought to better elucidate the role of hospital follow-up visits with a sample size that is relatively larger than most of those reported in the current literature.

Therefore, the aim of this study was to investigate a relatively large sample size to 1) determine whether ROM and SOI play important roles in the prediction of hospital readmissions; 2) identify other potential predictive risk factors of hospital readmissions; and 3) investigate the association between post-discharge hospital follow-up visits and readmissions.

## Methods

### Selection of participants

Data on all adult (18 years and older) patients admitted to a local urban tertiary care hospital (John Peter Smith Health Network, Fort Worth, TX, USA) during the period September 1, 2012 through June 30, 2015 were reviewed. Since this study focused on identifying independent risk factors associated with 30-day all-cause unplanned hospital readmissions after the index discharge, patients were either followed up 30 days after the index discharge or until they reached the endpoint of this study (i.e., June 30, 2015). Inclusion criteria: patients who had all-cause unplanned hospital readmissions within 30 days from the index discharge were included in this study. We allowed inclusion of patents with multiple readmissions if other study inclusion criteria were met. For those with multiple admissions, each admission was qualified as an index admission for subsequent readmissions. If there were more than one readmission within 30 days, we counted all readmissions where inclusion criteria were met. Exclusion criteria: Patients who were still in the hospital or had less than 30 days follow up at the endpoint of this study and those who had planned readmissions arranged upon index discharge were excluded from this study.

### Study design

This is a single center retrospective observation study. Data were extracted automatically from the electronic medical record. All eligible patients were divided into two groups (readmission versus no readmission). Basic patient demographics (age, sex, marital status, race, ethnicity, homeless status, and insurance coverage) and clinical variables (acute substance abuse upon index admission, history of psychiatric problems, ROM, SOI, length of stay in hospital, disposition, number of ICD-10 diagnoses, number of medications prescribed at time of index discharge, and number of hospital follow-up visits after index discharge) were analyzed and compared. Potential risk factors predictive of readmission were determined. Their accuracy and discrimination levels were compared. Among all hospital readmissions, the time interval in days between index discharge date and readmission date was measured and analyzed to determine association with readmission risk. In addition, all clinic visits were counted after the index discharge and prior to readmission. The time interval (number of days) between index discharge date and outpatient clinic follow up visit date (including Emergency Department [ED], Urgent Care Center [UCC], and other clinical visits) was also reviewed. The frequency of outpatient visits over time and the time interval to hospital readmission events were analyzed to determine the association with readmission risk. The local institutional review board approved this study and waived the individual informed consent requirement.

### Variables explanations

In this study, readmission refers to 30-day all-cause unplanned hospital readmission. Readmission days refers to the number of days between the index discharge date and date of readmission. At the index admission, we used the ICD-10 diagnostic codes for acute substance abuse, polysubstance abuse, substance intoxication with and without withdrawal symptoms, and intentional overdose from alcohol, stimulants (i.e. amphetamine/methamphetamine/cocaine), cannabis/THC, hallucinogens, sedatives, opioid use, and any other psychoactive substances such as K-2/Spice/ecstasy, etc. Patients admitted with psychiatric disturbances included those with anxiety spectrum disorders, bipolar spectrum disorders, major depressive disorder, psychotic disorders such as schizophrenia, schizoaffective disorders, those with suicidal ideation with a plan and means to complete a suicide, and those making a suicide attempt.

The number of medications prescribed at time of index discharge along with the number of discharge diagnoses were reviewed. It is assumed that patients with more chronic diseases might require more medications. In order to know whether healthcare providers prescribed enough medications for patients at time of discharge, a prescription ratio (the number of medications prescribed divided by the number of ICD-10 diagnoses at time of index discharge) was also measured. A high prescription ratio often indicates that enough medications were prescribed at time of patient index discharge. Whereas, a low prescription ratio indicates the number of medications prescribed is less than the number of discharged diagnoses. Examples further explaining this are included in the following conditions: 1) providers prescribed fewer medications thereby incompletely covering patient needs for their chronic diseases, or 2) patients might have enough medications at home due to their existing chronic diseases thereby not requiring any refill at time of index discharge. Though we are unable to determine the risk of medical non-compliance among these patients, a low prescription ratio may indicate fewer medications were prescribed at index discharge which might be related to inadequate management of chronic diseases in some of these patients.

### Data analysis

#### Continuous and categorical variables comparisons

Student’s t-tests and Wilcoxon rank-sum (Mann-Whitney) test were used to compare continuous variables between two groups, while Pearson Chi-square (*χ*2) analyses were used to compare categorical variables.

#### Kernel density estimation

Kernel density estimation (KDE) was used for frequency evaluation in patients with different readmission days’ intervals. Using this procedure allowed us to determine the function of random variables and to make general inferences about this patient population. When determining the association between hospital outpatient visits and readmissions, frequency lines were drawn and compared between groups with or without readmission by KDE.

#### Logistic regression analysis

In order to identify independent risk factors predictive of readmission and to avoid potential confounders, all potential variables were entered into a multivariate logistic regression model except outpatient visits. The association between hospital readmission and two different types of outpatient visits (i.e. ED/UCC and clinic visits) were determined separately in the following time-to-event analysis thus not included in the logistic regression. Variance inflation factor (VIF) was used for assessing the severity of multi-collinearity in the regression model and variables with high VIF (>10) were considered as having collinearity and were therefore excluded from regression analysis [32]. A Hosmer-Lemeshow test was performed to determine the goodness of fit for logistic regression.

#### Time-to-event (survival) analysis

The association between the two different types of outpatient visits (ED/UCC versus clinic visits) and readmissions was further determined by using time-to-event (survival) analysis with hazard ratios reported separately.

All descriptive and statistical analyses were performed using Stata 12.0 (College Station, TX). We used a 95 % confidence interval and a *p* value less than 0.05 was considered statistically significant.

## Results

**Fig. 1:**
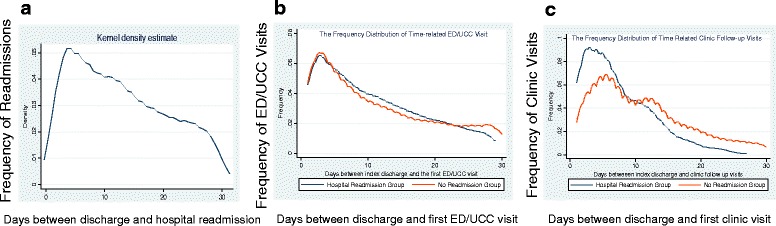
**a** Frequency of hospital readmissions within 30 days; **b** frequency of ED/UCC visits within 30 days (readmission versus no readmission group); **c** frequency of clinic visits within 30 days (readmission versus no readmission group)

There was a total of 55,887 hospital admissions during the study period. Among the 55,887 hospital admissions reviewed, 355 patients were excluded from this study including 83 patients who upon reaching the endpoint of this study, were still hospitalized. Another 272 admissions were excluded as a result of planned readmissions upon index discharge. Thus, 55,532 total admissions were eligible for final analyses representing 39,011 unique patients. We found an all-cause 30-day unplanned hospital readmission rate of 11 % (6011/55,532, see Appendix 1 Figure 1). Although, the average number of days between readmission and index discharge date was 12 (mean 12.9 ± 8.1 days and median 12 days), the peak time for readmission occurred within the first 7 days from index discharge with gradually decreasing readmission frequencies noted thereafter (Fig. 1a).

Table 1 details the demographic and clinical characteristics of samples between the ‘no readmission’ and ‘readmission’ groups. Significant group differences were found in the majority of variables. Patients who were readmitted within 30 days upon index discharge tended to be male, older, African American, homeless, and single in comparison with no readmission patients. Those patients had higher ROM and SOI determined by the APR-DRG and had longer hospitalizations during index admissions. We also found a slightly higher percentage of readmissions from patients who had Medicare and who were discharged directly to long-term or short-term nursing facilities (Table 1).

**Table 1:** Study patient characteristics

	Readmission No (*N* = 49,521)	Readmission Yes (*N* = 6011)
Demographics		
Mean Age --- year, (SD)***	44.5 (16.6)	49.3 (15.1)
Median Age --- year, (IQR)***	44 (30–57)	51 (38–60)
Male sex --- n, (%)***	19,367 (39)	3017 (50)
Marital status --- n, (%)***^c^		
Single --- n, (%)	23,435 (47)	3028 (50)
Married --- n, (%)	16,138 (33)	1560 (26)
Divorced --- n, (%)	4191 (8)	659 (11)
Race and ethnic group^c^		
Black --- n, (%)***	11,826 (24)	1907 (32)
White --- n, (%)	20,970 (42)	2489 (41)
Hispanic --- n, (%)***	18,444 (37)	1626 (27)
Clinical Variables		
Mean LOS -- days, (SD)***	5 (8)	7 (8)
Mean SOI --- n, (SD)***	2.1 (0.9)	2.6 (0.9)
Median SOI --- n, (IQR)***	2 (1–3)	3 (2–3)
Mean ROM --- n, (SD) ***	1.6 (0.9)	2.1 (0.9)
Median ROM --- n, (IQR)***	1 (1–2)	2 (1–3)
Disposition --- n, (%)***^c^		
Home	41,998 (85)	4887 (81)
Other facility^b^	3582 (7)	545 (9)
Insurance Payers --- n, (%)***^c^		
No insurance	9320 (19)	859 (14)
Medicare	7830 (16)	1251 (21)
Acute substance abuse	1999 (4)	257 (4)
History of psychiatric disorder(s)**	4296 (9)	593 (10)
Homeless***	1877 (4)	343 (6)
Primary care physician assignment --- n, (%)***	23,517 (47)	3593 (60)
Mean number of index discharge diagnoses --- n, (%)***	6 (6)	9 (7)
Median number of index discharge diagnoses --- n, (IQR)***	4 (1–9)	8 (4–13)
Mean number of medications prescribed upon index discharge --- n, (%)***	4 (4)	3 (4)
Median number of medications prescribed upon index discharge --- n, (IQR)***	3 (2–5)	2 (1–5)
Prescription Ratio --- (SD)***^a^	1.4 (1.5)	0.5 (0.9)
Prescription Ratio --- (median, IQR)***^a^	0.8 (0.3–2)	0.3 (0.1–0.7)
Prescription Ratio ≥ 0.5 --- (n, %)***^a^	32,325 (74)	2098 (45)
At least one ED/UCC/Clinic visit within 30 days after index discharge --- n, (%)***^d^	23,265 (47)	5699 (95)
At least one ED/UCC visit within 30 days from index discharge --- n, (%)***^d^	7232 (15)	5601 (93)
Days between first ED/UCC visit and index discharge (Mean, SD)***^d^	11.4 (8.7)	10.8 (7.8)
Days between first ED/UCC visit and index discharge (Median, IQR)^d^	9 (4–18)	9 (4–16)
At least one clinic visit within 30 days from index discharge --- n, (%)***^d^	20,218 (41)	2004 (33)
Days between first clinic visit and index discharge (Mean, SD)***^d^	10.9 (7.2)	7.3 (5.3)
Days between first clinic visit and index discharge (Median, IQR)***^d^	10 (5–15)	6 (3–11)

*Abbreviations*: *SD* standard deviation; *LOS* length of stay; *SOI* refers to All Patient Refined Diagnosis Related Groups Severity of Illness; *ROM* refers to All Patient Refined Diagnosis Related Groups Risk of Mortality; *IQR* interquartile range

**p < 0.01, ***p < 0.001

^a^Prescription Ratio refers to the number of medications prescribed upon index discharge divided by the number of index discharge diagnoses

^b^Other facilities includes: discharge to long term care, nursing, skilled nursing, short term hospital, and rehabilitation facilities

^c^Other groups divided under these categories were not shown in this table

^d^ED/UCC/Clinic only counts within 30 days after the index discharge (no readmission group) or prior to readmission (readmission group)

In addition, readmissions occurred more often in patients who had a greater number of discharge diagnoses but were prescribed fewer medications at time of index discharge. Particularly, when the prescription ratio was calculated, patients experiencing readmissions had significantly lower ratios than those not readmitted.

The multivariable regression model revealed that homeless, ROM, SOI, insurance coverage, and prescription ratio were strong independent risk factors that significantly predicted readmissions (Table 2). The adjusted Odds Ratio (OR) of homeless, SOI, and ROM were 1.35 (95 % CI 1.19–1.53), 1.31 (95 % CI 1.25–1.38), and 1.09 (95 % CI 1.05–1.14) separately while the adjusted ORs of insurance coverage and prescription ratio were 0.69 (95 % CI 0.63–0.74) and 0.33 (95 % CI 0.31–0.35). The C-statistics of the entire model was 0.73 which was reasonable discrimination for evaluating this risk prediction model. Additionally, the results of the Hosmer-Lemeshow goodness of fit test for logistic regression confirmed that the distribution fit the data well and the risk prediction was well calibrated (*χ* ^2^ = 1.68, *p* = 0.143).

**Table 2:** Odds ratios of different variables predictive of readmission

	Unadjusted odds ratio (95 % CI)	Saturated odds ratio (95 % CI)	Adjusted odds ratio (Final) (95 % CI)	VIF
Homeless	1.54 (1.36–1.73)	1.34 (1.12–1.61)	1.35 (1.19–1.53)	1.02
Severity of Illness	1.72 (1.67–1.77)	1.16 (1.09–1.24)	1.31 (1.25–1.38)	2.07
Risk of Mortality	1.59 (1.54–1.63)	1.16 (1.09–1.23)	1.09 (1.05–1.14)	2.17
No Insurance	0.72 (0.67–0.78)	0.70 (0.63–0.79)	0.69 (0.63–0.74)	1.05
Prescription Ratio ≥0.5	0.29 (0.27–0.31)	0.46 (0.43–0.50)	0.33 (0.31–0.35)	1.04
Age	1.02 (1.02–1.02)	0.99 (0.99–1.00)		
Gender	1.05 (1.00–1.10)	0.98 (0.91–1.06)		
In-hospital Length of Stay	1.03 (1.02–1.03)	1.01 (1.01–1.02)		
History of Psychiatric Disease	1.03 (1.01–1.06)	1.00 (1.00–1.00)		
History of Substance Abuse	1.06 (0.93–1.21)			
Final Model C-statistics			0.73	

Hosmer-Lemeshow goodness of fit test: *χ* ^2^ = 1.68, *p* = 0.143

*Abbreviation*: *CI* Confidence Interval

All adjusted odds ratios demonstrated statistical and clinical significance (*p* < 0.05)

In general, we found that the number of days between the initial hospital clinic visit and index discharge date in the readmission group was significantly fewer than in the no readmission group. There were no significant differences when their ED/UCC visits were compared (Table 1). Nearly 90 % (5381/6011) of readmissions were from ED/UCC visits. Thirty-two percent (1906/6011) of readmissions were experienced by patients having both ED/UCC and clinic visits. Among this group 88 % (1686/1906) were readmitted from the ED/UCC. Taken together, our findings showed significantly more readmissions were from subsequent ED/UCC visits after the index hospital discharge (Table 3, *p* < 0.001).

**Table 3:** Association of Outpatient Clinic Visits and Hospital Readmissions

	No ED/UCC/Clinic visits (*N* = 26,568)	ED/UCC visits only (*N* = 6742)	Clinic visits only (*N* = 16,131)	Both ED /UCC and Clinic visits (*N* = 6091)
No Readmission (*N* = 49,521)	26,256 (47.3 %)	3047 (5.5 %)	16,033 (28.9 %)	4185 (7.5 %)
Readmission from ED/UCC (*N* = 5381)	0	3695 (6.7 %)	0	1686 (3.0 %)
Readmission from Clinics (*N* = 318)	0	0	98 (0.2 %)	220 (0.4 %)
Readmission from others (*N* = 312)	312 (0.6 %)	0	0	0

Clinics: any outpatient visits within the first 30 days from index hospital discharge. Others: unknown readmission service or readmitted to other hospitals

*ED/UCC* Emergency Department or Urgent Care Center

The percentage in this table reflects the events/total 55,532 admissions *P* < 0.001

**Fig. 2:**
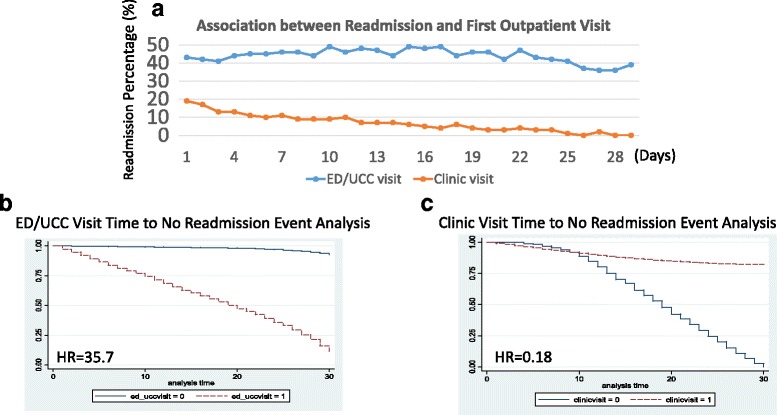
**a** Association between hospital readmission rates and first outpatient visits. No significant readmission changes noted among patients who had their initial ED/UCC visits at different time intervals from the index discharge. Readmission rates decreased with delayed interval to initial clinic visits. **b**/**c** Association between readmission and two types of outpatient clinics with their hazard ratios

Further detail investigations showed that the time interval between initial post-discharge clinic visit to the first subsequent ED/UCC visit was 8.1 ± 6.6 days (median 7 days). The peak time of ED/UCC visit occurred within 7–10 days from the index discharge date and followed the same pattern regardless of readmission (Fig. 1b). However, the peak time of clinic visit in the readmission group tended to be earlier than those not readmitted (Fig. 1c). Readmission rates were calculated separately to determine whether earlier clinic follow up impacted readmissions. Forty to fifty percent of the readmission rate occurred in patients who had ED/UCC visits regardless of the interval period from index discharge date, whereas readmissions were more frequent among patients experiencing earlier clinic visits after index discharge date (Fig. 2a). When time-to-event (outpatient visit time to no readmission event) analysis was performed, it showed that ED/UCC visits after the index discharge accounted for significant hospital readmissions where the calculated hazard ratio was 35.7 (95 % 30.6–41.5, *p* < 0.001, Fig. 2b), whereas clinic follow up visits did prevent patients from readmission only if clinic visits occurred after 10 days from index discharge date (hazard ratio = 0.18, 95 % CI 0.17–0.20, *p* < 0.001, Fig. 2c).

## Discussion

Hospital readmission rates are one of the leading quality measures describing patient care delivery. Thus, it is critically important to 1) identify high risk readmission factors, 2) develop strategic readmission prevention procedures, and 3) implement appropriate interventions among high-risk patients in a timely fashion [33]. Our readmission rate among all hospital admissions in this study was 11 % which is similar to others in the literature [34, 35]. Although the average interval from index discharge date to readmission was 12 days, the peak time occurred within the first 7–10 days indicating that readmission prevention measures should be in place as early as the first week after hospital discharge. Like Coller et al. [10] and Roberts et al. [21], we found that both ROM and SOI outcomes seem to predict readmission well along with other risks such as homeless status, no insurance coverage, and prescription ratio. Additionally, earlier clinic visits resulted in higher readmissions in this study indicating that simply arranging follow-up clinic visits does not prevent hospital readmission. Taken together, our study findings add some evidence to the current risk prediction pool of hospital readmission by validating the value of ROM and SOI, identifying peak time of hospital readmission, and determining the association between outpatient clinic visits and readmission in a relatively large sample size. These findings may provide guidance with respect to further implementation of effective readmission prevention interventions in the future.

Additionally, our results suggest that more attention should be paid to the prescription ratio at discharge since the available data shows that lower ratios occur among patients with a higher risk for readmission. This suggests that patients might not receive enough medications (new prescriptions and/or refills) at discharge. Some studies have emphasized proper medication adjustments and prescription refills in a timely manner; however, these have traditionally been limited to populations with high psychosocial risks which may not generalize to a broader patient population [36, 37]. Additionally, we found more readmissions occurred in homeless patients and that uninsured patients tend to experience fewer readmissions which are consistent with patterns observed by others [38–41].

Considering patients with higher SOI and ROM indices in the early outpatient clinic follow-up group, it is worthwhile to question whether or not adequate treatment was received during the index admission. Although the average LOS was greater in the readmission group, it is difficult to discern whether or not an additional 48-h of hospitalization would significantly improve the prognostic outcome (Table 1). Additionally, if patients were divided into groups by different SOI scores, the average LOS in high SOI patients was almost the same in both readmission and no readmission groups (see Appendix 2 Figure 2). As emphasis on hospital throughput metrics becomes more closely linked to the performance, quality, and reimbursement package of each healthcare provider, patients approaching the end of a predetermined LOS for a given diagnosis may be discharged prematurely to mitigate disincentives associated with failure to meet industry standards. In fact, Burke et al. [42] showed that the most common cause of preventable hospital readmission is incomplete management of index diagnoses. This suggests that the probability exists that inadequate treatment occurs during the index admission and is a credible risk factor influencing readmission.

Risks that affect hospital readmissions seem to be multi-factorial. Patient disease severity, psychosocial factors, healthcare access patterns, and the healthcare system itself should all be considered. Therefore, refocusing efforts on better recovery outcomes through health education and management before and during the discharge processes might prove to be invaluable in readmission reduction and avoidance. Standardized discharge processes using a more patient-centered approach consisting of patient health education, a comprehensive review of previous and newly added patient medications, careful messaging directed at individual patient best management strategies for their chronic disease(s), and augmenting hospital discharge clinics with community outreach programs or case/social manager follow-up within the first 7 days seems critically important to mitigate readmissions. Further determining high potential risks for hospital readmission among patients having post-discharge ED/UCC visits might also be helpful since the majority of readmissions were from these visits. Therefore, future prospective studies investigating additional related variables, focusing on patients with specific diseases, and implementing effective interventions for readmission prevention are warranted.

### Limitations

As a retrospective study using hospital admission data from a single urban publicly funded hospital, the methodology may have potential bias in terms of translation to other hospitals regarding accuracy of information, potential selection bias due to convenience sampling from one institutional database, and lack of comprehensive follow up data. We were unable to determine whether patient follow-up visits or hospital readmissions may have occurred in other hospitals after the index discharge. Risks that predict hospital readmission are multi-factorial and contributing factors unknown to us may have influenced our results. The prescription ratio might not be accurate enough to reflect the balance between comorbidity and patient compliance and should be used with caution. To our knowledge this is one of the larger sample size studies to examine a variety of clinical risk factors with strong potential to predict hospital readmissions. As such, external validity of the typical urban hospital patient population is well represented in our data as is ecological validity.

## Conclusion

Peak time for hospital readmission occurred within 7–10 days from the index discharge date. ROM and SOI determined by APR-DRG can be applied in general to predict relative risk of hospital readmission. Other variables such as patient insurance status and prescription ratio (the number of medications prescribed or refilled at time of index discharge divided by the number of discharge diagnoses) might be considered independent risks predictive of hospital readmission. Simply providing early post discharge follow up clinic visits appears insufficient to prevent hospital readmissions but more detailed study is needed to thoroughly answer this question.

## Figures and Tables

**Fig. 3:**
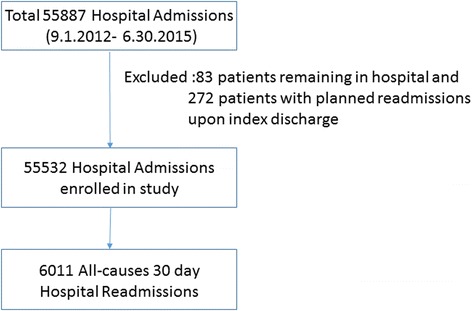
Flow diagram of study population

**Fig. 4:**
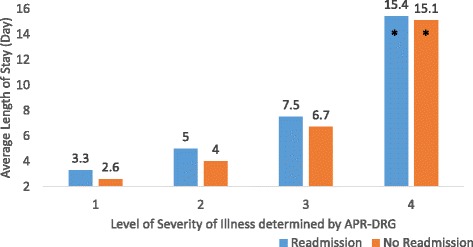
Association between hospital length of stay and level of severity of illness determined by APR-DRG (*p > 0.05 between two groups)
